# Clinical Impact of Patient-Specific 3D Models in Neonatal Surgery: A Case-Based Review of Applications and Future Directions

**DOI:** 10.3390/children12091202

**Published:** 2025-09-09

**Authors:** Oscar Girón-Vallejo, Bernardo Garcia-Nuñez, Isidoro Narbona-Arias, Alexander Siles-Hinojosa, Francisco Javier Murcia-Pascual, Moutasem Azzubi, Ignacio Gorriti, Dario Garcia-Calderon, Antonio Piñero-Madrona, Lucas Krauel

**Affiliations:** 1 Virgen de la Arrixaca Children’s Hospital, El Palmar, 30120 Murcia, Spainpineromadrona@gmail.com (A.P.-M.); 2 Corporació Sanitaria Parc Taulí, 08208 Barcelona, Spain; bgarcia@tauli.cat; 3 Regional University Hospital, 29010 Málaga, Spain; dr.narbona@gmail.com (I.N.-A.);; 4 Reina Sofía University Hospital, 14004 Córdoba, Spain; 5 King Abdullah Specialized Chidren’s Hospital, Riyadh 14611, Saudi Arabia; malzoubi@hotmail.com; 6 Department of Natural Sciences, Universidad Nacional de Rio Negro, Viedma R8500, Argentina; ignaciogorriti@cellams.com; 7 Pediatric Surgery and 3D4H Unit, SJD Barcelona Children’s Hospital, Universitat de Barcelona, 08950 Barcelona, Spain

**Keywords:** 3D modeling, neonatal surgery, surgical planning, patient-specific models, congenital malformations, oncology, medical imaging

## Abstract

Three-dimensional (3D) modeling and printing technologies are increasingly used in pediatric surgery, offering improved anatomical visualization, surgical planning, and personalized approaches to complex conditions. Compared to standard imaging, patient-specific 3D models—virtual or printed—provide a more intuitive spatial understanding of congenital anomalies, tumors, and vascular anomalies. This review compiles evidence from pediatric surgical fields including oncology, abdominal, and thoracic surgery, highlighting the clinical relevance of 3D applications. The technological workflow—from image segmentation to computer-aided design (CAD) modeling and multimaterial printing—is described, emphasizing accuracy, reproducibility, and integration into hospital systems. Several clinical cases are presented: neuroblastoma, cloacal malformation, conjoined twins, and two cases of congenital diaphragmatic hernia (one with congenital pulmonary airway malformation, CPAM). In each, 3D modeling enhanced anatomical clarity, increased surgeon confidence, and supported safer intraoperative decision-making. Models also improved communication with families and enabled effective multidisciplinary planning. Despite these advantages, challenges remain, such as production time, cost variability, and lack of standardization. Future directions include artificial intelligence-based automation, expanded use of virtual and mixed reality, and prospective validation studies in pediatric cohorts. Overall, 3D modeling represents a significant advance in pediatric precision surgery, with growing evidence supporting its safety, clinical utility, and educational value.

## 1. Introduction

Pediatric surgery faces unique anatomical, diagnostic, and technical challenges, stemming from structural variability, small body size, and the frequent presence of congenital malformations. Surgical procedures in newborns with congenital anomalies pose unique challenges due to structural size, anatomical variations and the high complexity of some surgeries. In this context, 3D modeling technologies for surgical planning have emerged as transformative tools in clinical practice, offering personalized anatomical models that may overcome the limitations of traditional two-dimensional imaging [1,2,3,4].

Over the past few years, the integration of 3D modeling and printing has steadily expanded across pediatric surgical specialties, including cardiac, oncologic, thoracic, and urologic disciplines. These models, generated from computed tomography (CT), magnetic resonance imaging (MRI), or three-dimensional echocardiography, enable intuitive visualization of complex structures, preoperative surgical simulation, and enhanced anatomical understanding by surgical teams, patients, and their families [5,6,7].

Numerous studies have documented successful clinical applications in complex congenital heart diseases, such as tetralogy of Fallot, single ventricles, and multiple septal defects [8,9,10,11]. In pediatric oncology, 3D planning has proven useful for retroperitoneal or thoracic solid tumors, improving the safe resection of masses in close relation to major vessels or deep structures [1,2,5,12]. Likewise, in neonatal thoracic surgery, printed models have been used to simulate thoracoscopic repairs (VATS) in cases such as esophageal atresia with tracheoesophageal fistula [13,14,15]. In pediatric surgery, the use of 3D technology in Spain dates back to 2015 in the field of pediatric oncologic surgery, with the first reported cases of neuroblastoma reconstructions for surgical planning [16]. In 2018, we also published our experience with the reconstruction of a bilateral Wilms tumor to quantify the preservation of residual renal tissue [17], as well as our experience with 3D technology as a planning tool in oncology, using it to locate pulmonary metastases and to practice surgery with different materials [18].

In some cases, 3D technology enables simulation of rare and high-risk scenarios, such as the EXIT (Ex Utero Intrapartum Treatment) procedure—a complex intervention performed while the baby remains partially within the uterus. These simulations are crucial for preparing surgical teams for high-risk, time-sensitive scenarios, enabling them to practice and refine their skills in a controlled environment before facing the real situation. The ability to recreate these rare situations with precision contributes significantly to improved outcomes and reduced intraoperative risk [1,19].

Beyond technical preparation, these tools have shown value in enhancing communication between clinicians and families, facilitating shared decision-making, and optimizing case comprehension among multidisciplinary teams [1,2]. Nevertheless, despite growing enthusiasm and adoption, structural limitations remain, including a lack of workflow standardization, cost variability, and the need for prospective studies to robustly validate clinical impact.

The aim of this review is to provide a critical and integrative perspective on the use of 3D technologies in a very specific field of pediatric surgery, such as neonatal surgery, examining its historical evolution, current clinical applications, practical knowledge from real cases and future directions in technological development and clinical validation.

## 2. Material and Methods

This article is structured as a narrative review, complemented by an illustrative case series drawn from clinical experience at our institution and partner centers. Literature was selected through targeted searches in PubMed and Scopus using keywords such as ‘3D modeling,’ ‘neonatal surgery,’ and ‘pediatric surgical planning.’ Priority was given to articles published between 2015 and 2024, including systematic reviews, original research, and technical reports relevant to pediatric applications of 3D models.

### 2.1. Technical Fundamentals of 3D Modeling in Neonatal Surgery

In order to establish a clear conceptual framework, three fundamental terms are defined below. ‘3D modeling’ refers to the creation of computational geometric representations of objects using computer-aided design (CAD) software. ‘3D printing’, or additive manufacturing, is the physical realization of such digital models through the controlled deposition of successive layers of material. ‘Virtual 3D model’ refers to a digital, interactive reconstruction of anatomical structures derived from medical imaging datasets. These models are displayed on screens or immersive environments (e.g., Virtual Reality or Augmented Reality) and allow users to manipulate spatial representations in real time for surgical planning, educational, or diagnostic purposes.

Developing a clinically useful 3D model for neonatal surgery is a highly specialized process that entails more than just generating 3D visualizations of medical scans. It consists of a chain of interdependent stages that integrate technical precision, specialized medical oversight, and advanced computational support. The final model must meet both anatomical fidelity and practical surgical utility.

The starting point, without exception, is the acquisition of high-quality medical imaging. Computed tomography (CT) and magnetic resonance imaging (MRI) have proven to be the most suitable modalities, depending on the anatomical structures to be reconstructed. CT is typically preferred for detailed evaluation of bone structures, the tracheobronchial tree, thoracic malformations, or vascular anomalies, due to its high spatial resolution and ability to obtain submillimetric isotropic slices [20,21]. In contrast, MRI offers irreplaceable advantages in soft tissue analysis—including fetal lung parenchyma, brain, visceral malformations, or pediatric abdominal tumors—because of its tissue characterization capabilities, absence of ionizing radiation, and superior soft-tissue contrast [9,22].

Once acquired—ideally with slice thicknesses of 0.5–1 mm and a 512 × 512 matrix—images are exported in DICOM format and undergo a rigorous process of cleaning and standardization. This includes reorienting data to orthogonal planes, harmonizing resolution across multimodal series (e.g., CT and MRI fusion), correcting motion artifacts, and removing acquisition distortions. In pediatric patients, particularly neonates or prenatal studies, this step is critical due to motion artifacts from respiration or fetal activity that may significantly degrade raw imaging data [22].

The next step—and arguably the most critical—is anatomical segmentation. This entails digitally isolating the structures of interest within the image volume, such as herniated contents in congenital diaphragmatic hernia, affected lobes in CPAM, or shared organs in conjoined twins. Segmentation is typically performed using semi-automated tools in specialized platforms such as 3D Slicer and Simpleware. Techniques include density thresholding, region growing, contour interpolation, and increasingly, artificial intelligence (AI) algorithms trained specifically on pediatric anatomy [23].

This stage follows a dual-logic approach: supervised AI models are employed to accelerate anatomical edge detection and minimize interobserver variability, while a multidisciplinary human team—including imaging technicians, biomedical engineers, and specialized radiologists—provides continuous oversight. This hybrid approach ensures high-quality segmentation, which is particularly vital in children and neonates, where anatomical proportions and spatial relationships differ substantially from adult cases.

Before the segmented model can be used clinically or surgically, it undergoes radiological validation. Unlike general educational simulations—where approximate representations are tolerable—high-complexity pediatric surgery demands millimetric accuracy. Therefore, segmented models are superimposed onto original images and reviewed structure-by-structure by expert radiologists. This validation workflow also integrates technical feedback, collaborative review, and AI-assisted alerts for morphological inconsistencies or residual distortions [23]. Only after passing this quality control phase is the model deemed suitable for surgical planning.

Subsequently, the clinical design phase begins. Segmented structures are converted into 3D meshes, typically in STL format, and undergo processes including mesh cleaning, smoothing, and topological optimization. At this stage, engineers resolve technical issues such as open meshes, virtual cut planes, and anatomical or surgical landmarking. The model is then prepared for 3D printing or virtual 3D modeling. This is performed within the proprietary Surgical Planner environment, an in-house platform that allows surgeons to visualize, manipulate, and simulate procedures on the virtual model prior to surgery.

Once validated, the surgeon may choose to use the model in its digital format or request physical printing. The choice of additive manufacturing technology depends on the anatomical context. For osseous structures, fused deposition modeling (FDM) is commonly used due to its affordability and structural definition. For soft tissues or complex combinations—such as visceral malformations or thoracoabdominal tumors—PolyJet printing is preferred for its multimaterial capability and variable rigidity and coloration [20,24]. In some cases, segmented-layer prints allow disassembly and reassembly, proving useful for training or family communication.

Alternatively, validated digital models are used directly in augmented or virtual reality platforms for preoperative simulation, surgical access planning, team training, or even intraoperative real-time overlay.

This workflow—from raw medical image to printed or virtual 3D model—is neither automated nor trivial. It requires coordinated effort from multiple experts: imaging technicians, validating radiologists, mesh-refining engineers, and surgeons simulating the procedure. While AI support is a key enabler for scaling this process, its success ultimately hinges on the quality of source data and clinical judgment. The true value of 3D modeling in high-complexity pediatric surgery lies in this balance between automation and expert oversight.

### 2.2. Interfacing with Surgical Teams: Real-Time Multidisciplinary Collaboration

What distinguishes the use of 3D modeling in pediatric surgery is not only the quality of the generated model but also its integration within a dynamic and functional clinical workflow. The success of this technology depends not solely on design precision but on the level of adoption, validation, and active use by the surgical team.

Integration typically begins with an initial multidisciplinary meeting involving radiologists, surgeons, anesthesiologists, neonatologists, and biomedical engineers. Together, they review medical images and define the clinical objectives of the model. Surgeons should actively participate in this process by working closely with radiologists, particularly in defining the specific anatomical regions that require segmentation. For example, in cases involving abdominal tumors, the bowels are typically not segmented, as they are displaced from the surgical field during the procedure. This step is essential to determine which structures must be represented, at what level of detail, and whether the model will serve diagnostic, simulation, communication, or direct surgical planning purposes.

This collaboration is formalized in “digital surgical review” sessions. The segmented 3D model is projected within the Surgical Planner environment and jointly analyzed by the care team. The surgeon can freely rotate the model, isolate structures, simulate cut planes, and plan the surgical approach in collaboration with all stakeholders. This dynamic, interactive process fosters deep, shared understanding of patient anatomy and results in safer, more personalized surgical strategies.

This approach has the potential to enhance clinical outcomes and redefines the surgeon’s role as the architect of operative strategy, enabling the simulation of multiple scenarios prior to the actual procedure. A clear example is the use of 3D modeling based on fetal magnetic resonance imaging to guide airway management during an ex utero intrapartum treatment (EXIT) procedure in a fetus with congenital high airway obstruction syndrome (CHAOS), as reported by Shalev [19]. The 3D-printed model, which included the mandible, tongue, larynx, and trachea, enabled the surgical team to simulate the intervention, select the appropriate instruments and reduce the time required to secure the neonatal airway—ultimately facilitating a successful intubation and an elective tracheostomy on the second day of life. This collaborative model has been successfully replicated in pediatric surgery.

Equally important, the modeling process is iterative and bidirectional. Throughout the workflow, the surgical team maintains continuous feedback with imaging and technical specialists, allowing iterative adjustments to the model. This two-way communication ensures that each case is approached as a customized clinical project, not a standardized software output.

In summary, 3D modeling does not replace clinical judgment or surgical experience; it amplifies both by transforming diagnostic images into manipulable, visual, tangible, and shareable representations. The model becomes not just a technical asset but a clinical dialogue space, a strategic rehearsal platform, and a catalyst for informed surgical decision-making.

### 2.3. Case Selection Criteria: When Is 3D Modeling Clinically Justified?

Despite widespread enthusiasm for 3D models in medicine, clinical implementation must be guided by well-defined criteria, especially in pediatric settings where resources are limited and clinical efficiency is paramount.

Certain pediatric cases involve congenital or acquired anomalies of such intrinsic anatomical complexity that two-dimensional imaging proves insufficient for accurate assessment. These include, for example, severe diaphragmatic hernias with intrathoracic liver herniation, retroperitoneal tumors encasing major vessels, or conjoined twins with fused vital organs. In such scenarios, three-dimensional reconstruction becomes indispensable for comprehending the true spatial relationships of the structures involved.

In the context of preoperative planning, particularly where sequential and technically demanding maneuvers are required, 3D models facilitate the virtual simulation of each procedural step. This is especially pertinent in neonatal surgery, where anatomical access is limited and the margin for error is exceptionally narrow. Such simulation enhances surgical preparedness and supports safer, more precise interventions.

Beyond their clinical utility, 3D models exert a substantial communicative and educational influence. Within pediatric practice, printed or digital reconstructions help elucidate complex diagnoses and surgical plans to parents or guardians, thereby improving informed consent and trust. Furthermore, these models serve as valuable pedagogical tools, offering residents, junior surgeons, and nursing teams an immersive understanding of intricate anatomy and surgical pathways in highly specialized settings [25].

Finally, in certain cases, 3D models have the capacity to directly influence surgical strategy. As demonstrated by Krauel et al., 3D modeling not only enhances surgical planning but also has a direct positive impact on safety, efficacy, and surgical outcomes in complex pediatric oncologic cases [16]. A parallel application in neonatal surgery allows for redefinition of incision sites, prediction of intraoperative challenges, and in some cases, the decision to avoid surgery altogether based on improved anatomical insight.

The use of 3D modeling should not be universal or indiscriminate. Each case must be evaluated individually, weighing expected clinical benefits against the technical and logistical effort required. However, as the technology matures and production times and costs decrease, 3D modeling continues to expand as a component of increasingly personalized, precise, and visually intuitive medicine [26].

## 3. Clinical Cases

We present illustrative cases in which 3D models were utilized for pathologies diagnosed during the neonatal period (Table 1). In some of these cases, surgery was performed during the infancy period due to surgical scheduling constraints or clinical indications. Nevertheless, as these are procedures that can be performed during the neonatal stage and, in our cases, were performed in patients around one year of age, we consider them of interest and worthy of inclusion.

*Case 1: Diaphragmatic hernia*

A male fetus was diagnosed at 20 weeks of gestation with a right-sided congenital diaphragmatic hernia (CDH) during routine prenatal ultrasound. Findings included a persistent right umbilical vein and non-visualization of the gallbladder. Fetal MRI confirmed herniation of liver segments VII and VIII and the gallbladder through a posterolateral diaphragmatic defect. Genetic testing, including karyotype, chromosomal microarray, and targeted exome sequencing for CDH-related genes, showed no abnormalities. These findings defined the case as anatomically complex, carrying a poorer prognosis.

The perinatal survival prognosis was based on the most recognized and widely supported parameters in the literature, such as the lung-to-head ratio (LHR) and sidedness. Poor prognosis criteria included the right-sided location and liver herniation, as well as an unfavorable LHR. However, the ultrasound examination revealed residual right lung parenchyma and contralateral lung that provided a more hopeful impression than strictly derived from these parameters.

**Figure 1:**
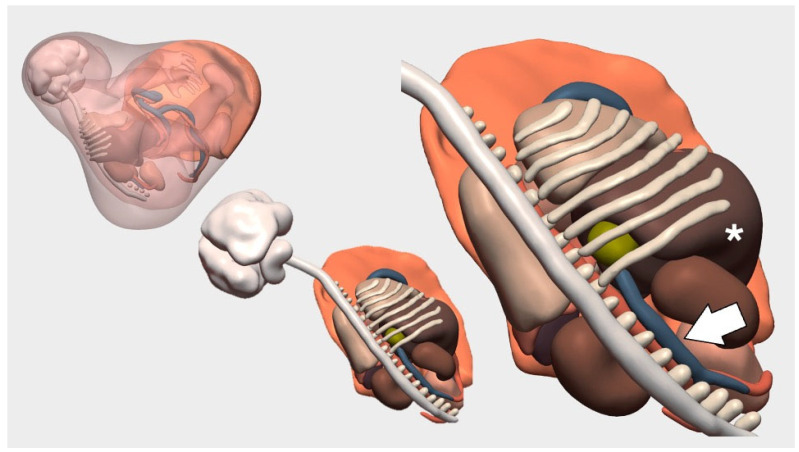
Virtual 3D reconstruction of right-sided diaphragmatic hernia based on fetal MRI. The different fetal planes are visualized and can be selectively removed in the Surgical Planner to better assess the dimensions of the diaphragmatic hernia. The liver is marked with an asterisk, and next to it, the infrahepatic vena cava can be seen (white arrow).

After counseling and the family’s decision to continue with the pregnancy, the option was offered to complete the evaluation at a more advanced gestational age using ultrasound and MRI. As an additional prognostic factor, a novel method was used to estimate total fetal lung volume using a high-fidelity 3D virtual model created by fusing images from both techniques (Figure 1). At 32 weeks, this second fetal MRI was performed, enabling the generation of the 3D model. This approach not only offered a more hopeful assessment of total lung volume compared to previous prognostic parameters but also provided a detailed spatial understanding of the defect and herniated structures, reinforcing the feasibility of primary repair. Moreover, it facilitated interdisciplinary coordination and helped the family visualize and better understand the anatomical defect, thus promoting informed decision-making.

The newborn was delivered at 40 + 2 weeks via induced vaginal birth, weighing 3900 g, and underwent surgical repair at 48 h of life through a right posterolateral thoracotomy. Herniated liver segments, the gallbladder, and small bowel loops were reduced, and a straightforward, tension-free primary closure was successfully achieved without prosthetic material.

The postoperative recovery was smooth, and the newborn was discharged in good condition on postoperative day 7 after early extubation.

This case illustrates the value of 3D virtual modeling—based on fused MRI and ultrasound images—in complex right-sided CDH. Beyond offering refined prognostic information, this technology enhances preoperative planning, strengthens interdisciplinary coordination, and fosters meaningful parental counseling. Ultimately, it empowers both clinicians and families to face these challenging prenatal diagnoses with clarity and confidence.

*Case 2: Diaphragmatic hernia with CPAM (Congenital Pulmonary Airway Malformation)*

A 12 weeks’ gestation fetus was identified via prenatal ultrasound with a left thoracic lesion that progressively enlarged, eventually occupying the entire hemithorax, causing significant displacement of the heart and mediastinum to the right, and strongly suggesting contralateral pulmonary hypoplasia. The situation was further complicated by the presence of a congenital diaphragmatic hernia, an extraordinarily rare combination, especially evident in the prenatal period. The CPAM volume ratio (CVR) was 2.1 and the lung-to-head ratio (LHR) was 0.8, both considered unfavorable prognostic indicators.

**Figure 2:**
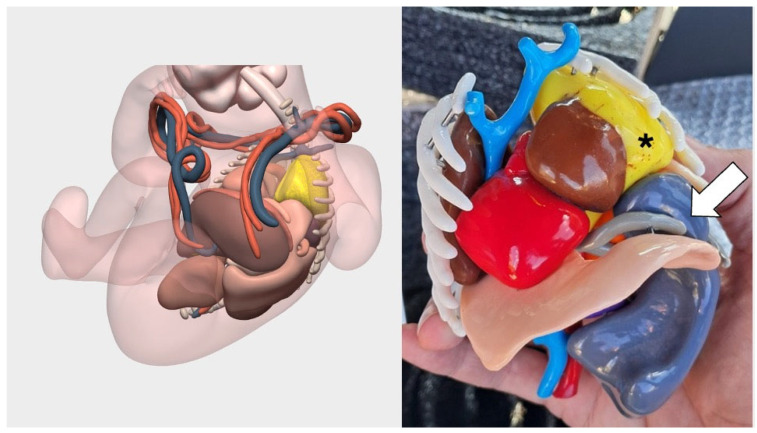
Case of left-sided diaphragmatic hernia with congenital pulmonary airway malformation (CPAM). Virtual reconstruction based on fetal MRI and 1:1 scale printed model. The CPAM is in yellow and marked with asterisk. The white arrow indicates the diaphragmatic defect.

**Figure 3:**
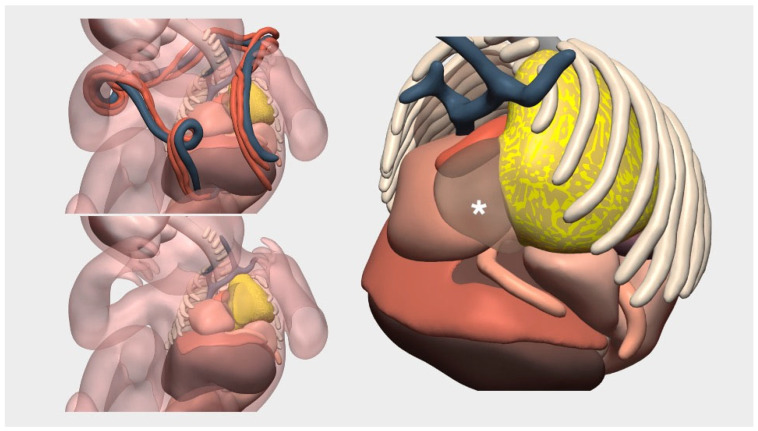
**Three-dimensional** virtual model of left-sided diaphragmatic hernia with congenital pulmonary airway malformation (CPAM). Surgical Planner allows us to add and remove layers of the virtual 3D model: in this case, the umbilical vessels have been removed. In the third image, the diaphragmatic hernia defect is visualized from a cranial view. The CPAM is in yellow and the normal lung is marked with asterisk.

Given the exceptional complexity of the case, the team decided early in the pregnancy to complement planning with a three-dimensional model. Upon detecting the malformation’s progression during the second-trimester ultrasound and recognizing both the rarity and massive extent of the CPAM (Congenital Pulmonary Airway Malformation), the team opted to create a 3D-printed model (Figure 2). This proactive approach aimed to ensure the best possible preparation for an emergency postnatal surgery or even an EXIT (Ex Utero Intrapartum Treatment) procedure, if necessary. High-resolution fetal imaging was used to produce patient-specific anatomical reconstructions at life size, available in both digital and physical formats (Figure 3).

Because the model was printed at a 1:1 scale, it closely resembled the actual anatomy encountered during surgery. This high degree of anatomical accuracy enabled improved surgical and spatial planning, as well as physical simulations, and ultimately contributed to reducing operative time by anticipating the major technical challenges.

The 3D models allowed both the surgical and obstetric teams to visualize, assess, and simulate the complex anatomical relationships prior to birth, transforming abstract imaging into a concrete surgical guide. This early, detailed spatial understanding facilitated precise planning for both cesarean delivery and postnatal surgical interventions.

The birth was scheduled by cesarean section. The newborn presented with good Apgar scores and an approximate birth weight of 2890 g. The diaphragmatic hernia was repaired via thoracotomy within the first 48 h of life. Postoperative recovery was excellent, and the patient was discharged on postoperative day seven. As per our institutional protocol, resection of the CPAM is planned around 9 months of age. Delaying CPAM resection made it possible to avoid an excessively prolonged neonatal surgery and, at the same time, to promote compensatory growth of the contralateral hypoplastic lung.

*Case 3: Thoraco-omphalopagus conjoined twins*

**Figure 4:**
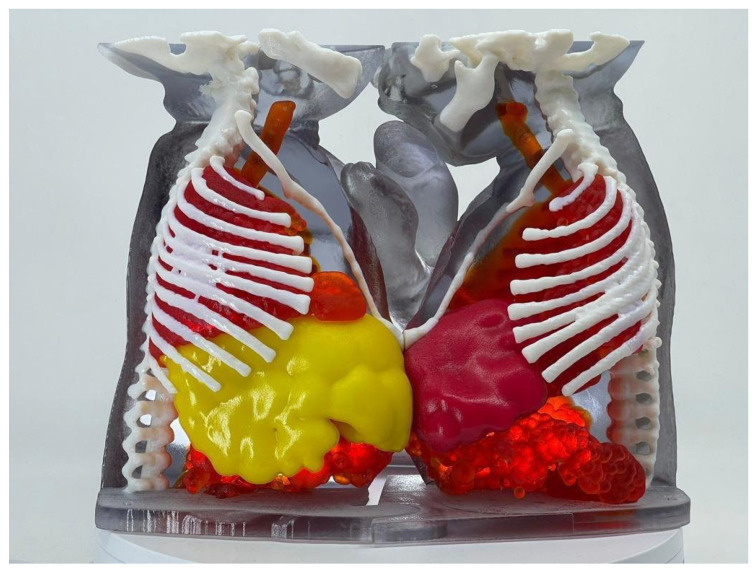
3D-printed replica of a case of thoraco-omphalopagus conjoined twins.

A pair of thoraco-omphalopagus conjoined twins, joined at the lower thorax and upper abdomen with partial hepatic parenchymal fusion and a shared abdominal wall, underwent detailed evaluation for potential surgical separation. Although the anatomical connection was relatively limited, three-dimensional (3D) surgical planning and physical modeling proved to be essential tools in optimizing the multidisciplinary surgical approach. High-resolution diagnostic imaging was obtained through fetal magnetic resonance imaging (MRI), enabling the generation of high-fidelity 3D digital models that accurately represented the fused anatomy, including the intrahepatic vascular distribution and spatial relationships of adjacent structures (Figure 4).

**Figure 5:**
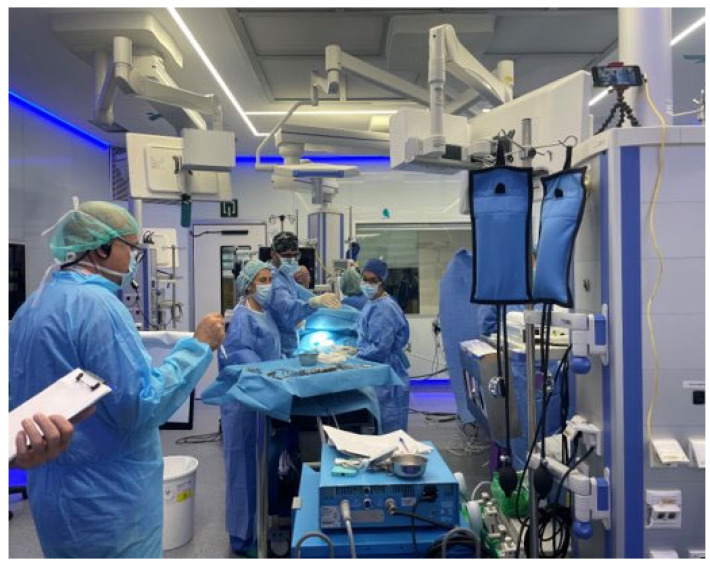
Teamwork simulation at the OR prior to surgery. In the foreground, the team leader directs the actions of the entire team. In this simulation, the 3D-printed model was used to coordinate the work of the whole medical–surgical team.

These 3D digital reconstructions not only facilitated detailed preoperative anatomical visualization, but their conversion into life-size 3D-printed physical replicas also allowed for comprehensive procedural simulation. This simulation encompassed the logistical planning of synchronized anesthesia induction, intubation strategy, precise dual-table patient positioning, and the stepwise execution of the surgical plan. Furthermore, it enabled rehearsal and coordination between two multidisciplinary surgical teams operating in parallel (Figure 5). This preparatory process significantly enhanced team coordination, minimized intraoperative uncertainty, and played a decisive role in achieving a safe, efficient separation with excellent postoperative outcomes.

*Case 4: Craniopagus conjoined twins*

The surgical separation of craniopagus twins represents one of the most complex challenges in pediatric neurosurgery.

Conjoined twins were diagnosed with total vertical craniopagus, one of the rarest and most complex forms of cranial conjoining. They were fused at the vertex of the skull with an asymmetrical angular cranial fusion, which posed significant surgical challenges. Advanced imaging revealed extensive calvarial fusion, a complex shared dural venous system, and, critically, interconnected cerebral parenchyma with areas of true cortical fusion.

This degree of cortical interdigitation and parenchymal bridging significantly increased the complexity of the case, necessitating meticulous planning to minimize neurological risk and preserve functional brain regions.

A four-stage surgical plan was performed, including the placement of tissue expanders several months prior to the final stage to facilitate soft tissue coverage and scalp closure.

**Figure 6:**
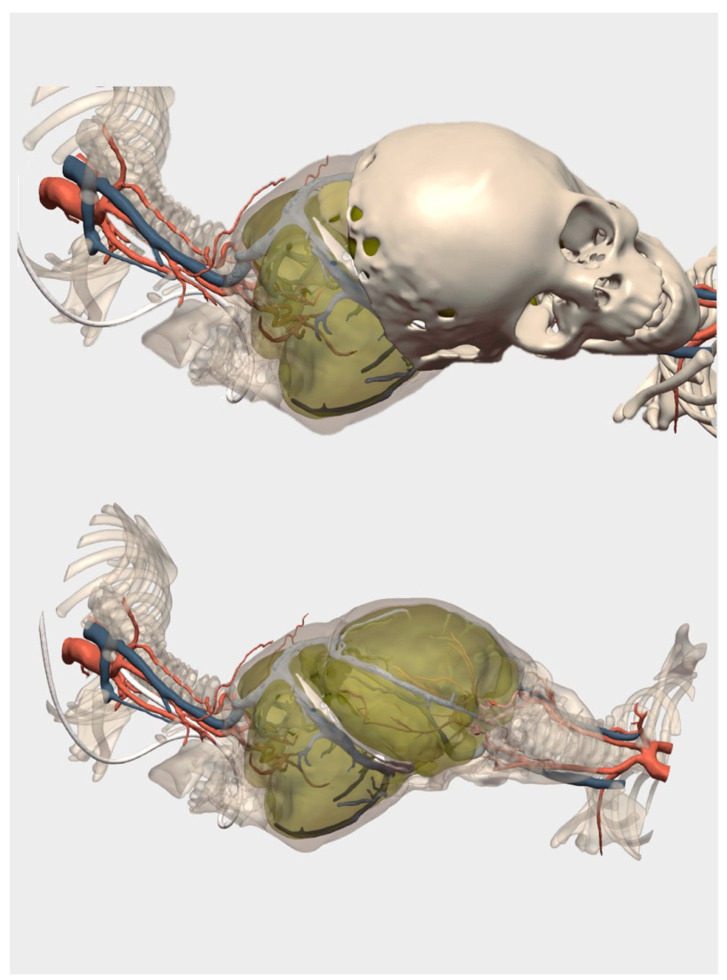
**Three-dimensional** virtual model of craniopagus conjoined twins.

High-resolution 3D virtual and printed models were developed, allowing precise visualization of the angulated cranial interface and detailed mapping of the shared and separate dural venous anatomy (Figure 6). These models enabled simulation of venous disconnection, cranial osteotomies, as well as identification of cortical fusion zones to guide safe parenchymal dissection.

Moreover, the use of 3D modeling enhanced multidisciplinary collaboration among neurosurgery, craniofacial plastic surgery, anesthesiology, and intensive care teams.

The final separation procedure lasted over 18 h and was successfully completed, with favorable neurological outcomes for both twins.

*Case 5: Cloacal malformation reconstruction*

Cloaca represents the most complex end of the spectrum of anorectal malformations in female patients. A detailed understanding of the anatomy, as well as the spatial relationship between the various structures and the pelvis, is crucial for successful repair in these surgically challenging cases, despite definitive intervention typically occurring during infancy.

**Figure 7:**
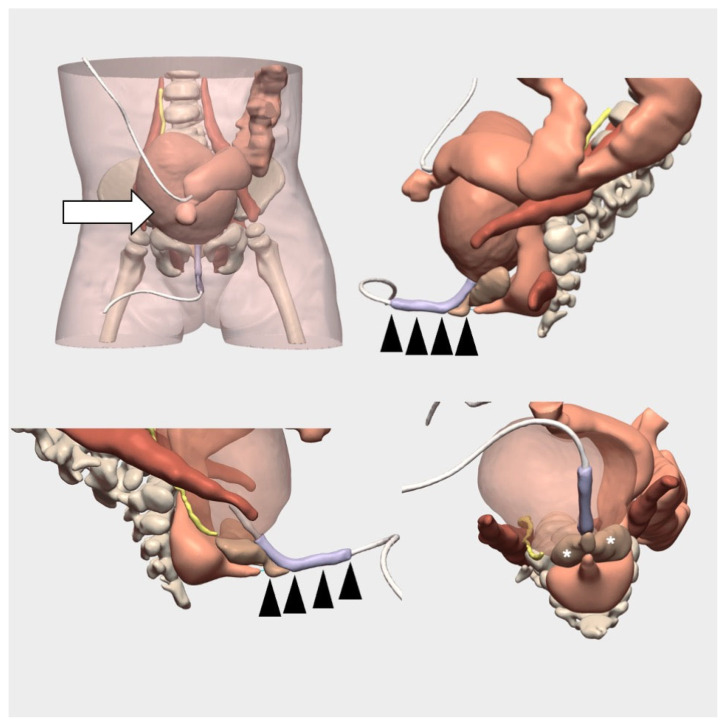
**Three-dimensional** virtual cloacagram for cloacal malformation reconstruction. It allows for multiple perspectives as well as measurement of the common channel and the urethra. Colostomy (white arrow, upper left), common channel (black arrowheads, upper right and lower left), and hemivaginas (asterisks, lower right).

An 18-month-old female infant underwent cysto-vaginoscopy under general anaesthesia to place a urinary catheter using an 8 Fr Foley catheter, and another Foley catheter was positioned in the mucosal fistula. A cloacogram via CT scan with 3D reconstruction was subsequently performed (Figure 7). The bladder was filled with 5% diluted water-soluble contrast in saline, followed by balloon deflation to evaluate the voiding phase. Contrast medium was also introduced through the distal colostomy to perform a colostogram.

During the 3D cloacogram, retrograde filling of both hemivaginas was observed. An ectopic right ureter draining into the bladder neck was identified, along with two hemivaginas and two hemiuteri, findings previously noted on cysto-vaginoscopy. Urethral length was measured at 1.8 cm, the common channel length at 2.6 cm, and the distance from the rectal fistula to the perineum was 3 cm. Additionally, a grade II vesicoureteral reflux and a fusion defect of the L3-L4 lumbar vertebrae were observed.

Based on the imaging findings, total urogenital sinus mobilisation (TUM) was selected as the surgical technique of choice for this patient.

The use of cloacogram and 3D reconstruction allowed for a more accurate assessment of cloacal anatomy and facilitated the selection of the most appropriate surgical approach, with the aim of achieving optimal postoperative outcomes.

*Case 6: Bilateral neuroblastoma*

Although this is not strictly a surgery performed during the neonatal period, this case serves to illustrate the importance of a 3D model applied to neonatal diagnosis. A 22-day-old newborn presented with vomiting and constipation since birth, along with a right-sided Horner’s syndrome. Ultrasound revealed bilateral adrenal lesions extending to the midline. A biopsy was performed, and the diagnosis was bilateral Ms neuroblastoma with liver and bone marrow metastases.

During the course of the disease, at 6 months of age, the patient developed intractable diarrhea due to VIP secretion, which could not be controlled in the Intensive Care Unit, necessitating earlier-than-planned surgical excision.

**Figure 8:**
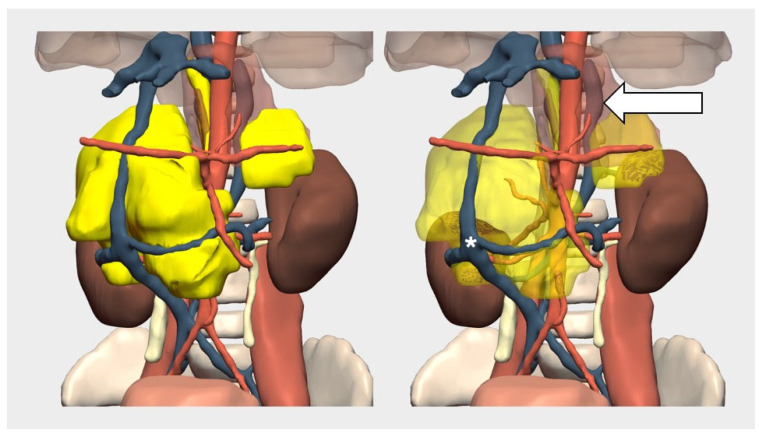
**Three-dimensional** virtual model of bilateral neuroblastoma. Tumor (yellow) can be made more transparent to visualize the vascular structures it surrounds. The white arrow indicates a hemiazygos vein dilated by tumor compression. The asterisk marks the inferior vena cava.

In this case, a 3D model was created to assess the involvement of major vessels, which confirmed the encasement of the aorta and both right and left renal arteries and veins, as well as an anterior displacement of the vena cava (Figure 8). Surgery achieved an approximately 98% resection.

Histopathological analysis showed that the left-sided component consisted of mature neuroblastic tissue, whereas the right-sided component had a worse prognosis histology, with N-myc amplification on that side. As a result, the patient was treated with the high-risk SIOPEN protocol.

During follow-up, the disease progressed, prompting reassessment with MIBG, which revealed uptake in a right retrocrural component in the posterior mediastinum that had gone unnoticed during surgery. However, this component had already been visible in the initial 3D model but was overlooked during the intervention. The 3D model also enabled the evaluation of the potential origin of the recurrence and the planning of a mediastinal surgical approach to resect it. The patient is currently undergoing treatment with dinutuximab, pending surgery for the residual tumor.

## 4. Discussion

The use of patient-specific 3D models in neonatal surgery has evolved from a technological novelty into a clinically valuable tool, supported by growing evidence across surgical specialties. Although much of the literature originates from referral centers with dedicated platforms and staff, our experience aligns with reported data, highlighting consistent patterns that confirm the strategic and clinical benefits of this technology.

In pediatric oncology, 3D modeling has proven useful for retroperitoneal, mediastinal, and renal tumors involving critical structures. Valls-Esteve et al. showed that anatomical reconstruction enables safer resections and supports organ-sparing decisions, particularly in bilateral Wilms tumors [12,17]. This approach allows volumetric estimation of renal parenchyma, essential for assessing bilateral partial nephrectomy feasibility without dialysis. Literature indicates that 3D planning can lower the risk of postoperative renal insufficiency, a crucial functional outcome [2,21]. In 2018, we reported our experience using 3D reconstruction in nephron-sparing surgery for bilateral Wilms tumor, which enabled preoperative estimation of residual renal parenchyma [17].

Congenital heart surgery was among the first fields to adopt 3D modeling for complex anomalies. Francoisse et al. identified several scenarios where 3D printing shaped surgical strategy, particularly in ventricular or arterial malformations [2]. Similarly, Ghosh et al. showed that 3D models influenced technique choice and reduced bypass time [8].

In neonatal thoracic surgery, Hong et al. demonstrated that 3D-printed models improve planning of minimally invasive procedures, optimizing trocar positioning, anticipating access limitations, and rehearsing anastomosis [14]. This is particularly valuable in neonates, where minimal anatomical variation can lead to major complications.

Even in emergencies, such as EXIT procedures, 3D modeling has guided decision-making. VanKoevering et al. reported the first prenatal 3D printing of fetal maxillofacial anatomy, which avoided an unnecessary EXIT by confirming airway patency [27].

Overall, evidence validates the utility of 3D models across pediatric subspecialties, with clear impact on reducing complications, optimizing operative time, and enabling safer surgical decisions [2,8,28].

### 4.1. Educational and Communicational Impact

One of the most important—and often underestimated—contributions of 3D modeling in pediatric medicine lies not only in technical precision or operative efficiency, but in its ability to transform educational processes, communication, and shared decision-making between medical teams, patients, and families.

This impact transcends the surgical field and redefines how we understand, explain, and teach anatomy, pathology, and therapy in high-complexity scenarios.

#### 4.1.1. Surgical Education and Interdisciplinary Training

In surgical education—particularly in neonatal surgery—exposure to real cases with rare pathologies or complex anatomical variants is limited. The incorporation of 3D-printed models faithful to the patient’s specific anatomy offers a simulation environment that far surpasses traditional teaching tools like atlases or generic models [7].

In our cases, models were used in preoperative sessions involving pediatric surgeons, anesthesiologists, intensivists, residents, and nurses. These joint reviews allowed alignment of strategies, anticipation of complications, and rehearsal of surgical approaches before entering the operating room. This dynamic mirrors what Pratt et al. describe in fetal surgery, where 3D models not only improve anatomical understanding but also strengthen teamwork and integrated surgical preparation [22].

In procedures such as double outlet right ventricle correction or bilateral partial nephrectomy, physical models enable spatial planning of surgical access, feasibility of resection, and anticipation of intraoperative adjustments. This spatial intelligence is particularly valuable in surgical training, where understanding 3D topography is difficult to acquire through 2D techniques [2,8].

Moreover, in minimally invasive contexts like neonatal VATS, rehearsing on printed models allows surgeons to practice dissection, anastomosis, or resection under nearly identical conditions to reality. Hong et al. show how 3D-printed thoracic models allow precise trocar placement planning, simulating distances, angles, and safety margins, directly reducing errors and operative time [14].

#### 4.1.2. Physician–Parent Communication and Informed Consent

In pediatrics, informed consent presents unique challenges: the patient does not decide, and guardians—usually the parents—face steep learning curves in understanding complex medical information, especially regarding congenital or oncologic conditions.

Here, 3D models are invaluable communication tools. Unlike traditional diagrams or verbal descriptions, showing parents their child’s exact anatomy and pathology transforms clinical interaction. It clarifies the surgical procedure, transparently communicates risk, and builds trust in the decision-making process [2,25].

Francoisse et al. documented that the use of physical models in congenital heart consultations improved parental understanding, increased perceived control, and reduced preoperative anxiety [2]. In emotionally charged contexts like infant cardiac surgery, this benefit is hard to quantify but extremely relevant. Similarly, in pediatric oncology, modeling improves informed consent quality and enhances family engagement with the therapeutic plan [20,21,25].

#### 4.1.3. Application in Rare or High-Stress Scenarios

Some clinical situations elevate the communicational and educational value of 3D models even further. The EXIT procedure is one such example: a highly complex surgery performed at birth with the fetus partially delivered while maintaining placental circulation. The decision requires careful multidisciplinary evaluation and must be clearly understood and accepted by the parents.

VanKoevering et al. describe the use of prenatal 3D modeling to assess airway obstruction in a fetus with maxillofacial malformation [27]. The model excluded the need for EXIT, sparing the mother a high-risk procedure. This case illustrates how 3D modeling can explain highly complex clinical scenarios to pregnant mothers with a clarity that 2D ultrasound or MRI cannot achieve. In situations with zero margin for error and maximum parental anxiety, this added value is invaluable.

Shalev reported the first documented use of 3D modeling and printing of the fetal airway as a preoperative planning tool for an ex utero intrapartum treatment (EXIT) procedure in a fetus diagnosed with congenital high airway obstruction syndrome (CHAOS) caused by a large cervical lymphatic malformation [19]. Using fetal MRI, key anatomical structures—including the mandible, tongue, larynx, trachea, and the mass—were segmented and printed separately to reconstruct a precise 3D model of the airway. This model allowed the multidisciplinary team to simulate various airway management scenarios, select appropriate equipment, and anticipate potential challenges.

The 3D model was instrumental in developing a detailed airway strategy, enabling rapid and successful intubation during the EXIT procedure, while the fetus remained partially delivered and oxygenated via the placenta. A planned tracheostomy was performed on the second day of life. Despite technical limitations such as lower resolution of MRI compared to CT and potential fetal movement artifacts, the use of the model improved team coordination and decision-making. The case illustrates how prenatal 3D modeling can enhance perinatal planning, reduce risks, and potentially become a valuable tool for determining the necessity and approach of EXIT procedures in complex fetal airway obstructions.

#### 4.1.4. New Directions: Communicational Tools in Digital Health

As 3D modeling expands into virtual environments (AR, MR, holography), new opportunities for clinical communication arise. Some teams are beginning to use interactive virtual models on tablets or XR devices to explain procedures to families or facilitate interdisciplinary discussions. While not yet mainstream, this format holds immense promise for improving communicational accessibility [14,25].

In the near future, such tools may integrate into electronic health records, allowing families to access interactive 3D representations of their child’s anatomy from home, complete with visual explanations of the proposed surgery. This evolution represents not just a technical leap, but a cultural shift in how we understand participatory medicine and health education.

### 4.2. Technical, Logistical, and Cost Considerations

The implementation of 3D modeling in neonatal surgery depends not only on technological availability but also on a set of technical, logistical, institutional, and economic conditions that define its clinical feasibility. Unlike other medical technologies, 3D modeling is not a closed device but rather a process composed of multiple stages—imaging, segmentation, modeling, validation, printing, or virtual visualization—that require coordination among multiple stakeholders and resources. This section addresses those complexities, highlighting both strengths and practical limitations observed in our experience and in the literature.

#### 4.2.1. Dependence on Medical Image Quality

The first critical link in the chain is image acquisition. Without high-quality medical imaging—with thin slices and optimized protocols—3D modeling loses precision and clinical value. In our experience, all reconstructions were based on high-resolution CT (helical multidetector CT with slice thickness < 1 mm) or MRI with specific sequences such as high-resolution T2 or volumetric HASTE and SSFSE in fetal or neonatal contexts.

As noted by Pratt and Punyaratabandhu, model accuracy depends directly on the resolution of the original DICOM images, their ability to delineate anatomical borders, and tissue contrast [20,22].

#### 4.2.2. Segmentation: The Operational Bottleneck

Segmentation is perhaps the most critical and demanding step in the workflow. This process requires clinical expertise, knowledge of pediatric anatomy, and mastery of specialized software. In our experience, collaboration between specialized radiologists and modeling specialists was essential to achieving accurate segmentation. As noted by Pratt et al., most published cases in fetal and pediatric surgery relied on manual segmentation with second-operator validation, since automation still struggles with fetal motion and small vessels [22].

This bottleneck not only consumes time (2–8 h per model depending on complexity) but also represents the most error-prone step. Cross-validation and feedback between the modeler and surgical team are essential before proceeding to printing or simulation.

#### 4.2.3. Physical Printing vs. Virtual Modeling

A major logistical decision in 3D modeling is whether to print the model or use it in virtual environments (AR, VR, screen-based visualization). Both approaches have advantages and limitations.

In our cases, physical printing—PolyJet for cardiac models and SLA for abdominal and thoracic structures—was chosen for all patients, with the exception of the bilateral neuroblastoma case and the cloacal malformation. This decision was based on the need for tactile interaction by the surgical team and the utility of the model in family communication. However, we recognize that in some cases—vascular planning or intraoperative simulation—virtual models may be equally or more effective, faster, and more economical.

Hong et al. report that virtual models in neonatal thoracic surgery simulators allow repeated rehearsals without physical wear and costly reprints [14]. Mixed-reality platforms are emerging, enabling 3D model manipulation on tablets or HoloLens-type devices, opening new possibilities for remote and multidisciplinary access.

#### 4.2.4. Production Time and Institutional Logistics

From image acquisition to printed model delivery, turnaround time ranged from 48 to 96 h. This depends on model type, number of segmented structures, surgical validation, and printer availability. In urgent care settings, such as neonatal surgery or decompensated cardiac cases, this may be a significant limitation.

For this reason, some institutions have begun producing “rapid” planning models without physical printing, using open tools like 3D Slicer or AR platforms that deliver validated digital models within 24 h [8,22]. However, this requires not only technical capabilities but also trained personnel, protected time, and institutional support.

In-house medical 3D printing labs—such as those described by Rybicki in the U.S. and Europe—have proven effective in reducing cost and turnaround [29]. These centers centralize acquisition, segmentation, and manufacturing, and standardize quality control. For institutions lacking such infrastructure, external partnerships provide a viable, though more dependent, alternative.

#### 4.2.5. Cost and Sustainability

The cost of producing a 3D model varies according to technology, size, number of structures, printing method, and level of detail. In our experience, model costs ranged from €350 to €1000. Virtual-only models, depending on processing complexity and software platforms, generally cost significantly less—typically between €100 and €300—since they avoid material and printing labor costs. These values are consistent with international reports [2,20].

While the cost may seem high, it should be evaluated within a cost-effectiveness framework. Publications have shown that 3D models can

– Reduce operative time by 15–30% in orthopedic oncology, particularly in the treatment of osteosarcoma [20,21].
– Lower intraoperative complication rates [2]
– Shorten postoperative length of stay in pheochromocytoma and paraganglioma surgery [28]
– Avoid unnecessary procedures such as EXIT in selected cases [27]

Additionally, improvements in family decision-making and surgical team training provide indirect institutional value [25].

Some hospitals have adopted mixed funding schemes: pooling budgets from surgery, radiology, education, and innovation, integrating modeling into clinical pathways without relying on individual case billing [22].

### 4.3. Limitations and Future Perspectives

Despite growing international enthusiasm, significant barriers remain on the path to systematic, equitable, and clinically validated implementation of 3D technology in pediatric surgery. The primary technical challenge is image quality. Pediatric imaging is subject to motion, sedation needs, and variable anatomy across developmental stages, complicating acquisition of clean, reproducible multiplanar data. As discussed earlier, segmentation is time-intensive, operator-dependent, and lacks international standards. Despite progress in AI, automated segmentation for pediatric structures—especially fetal soft tissues and small vessels—still shows significant error rates. Many centers also lack the hardware for advanced volume rendering, multimaterial printing, or immersive 3D interaction. This technological gap contributes to major inequities between high-resource academic hospitals and second- or third-tier facilities.

Despite the promising applications described, it is important to acknowledge the current limitations of the available evidence. Most studies to date are based on retrospective case series or single-center experiences, often lacking control groups or standardized outcome measures. This methodological heterogeneity restricts the generalizability of findings and underscores the need for high-quality research. In particular, there is a scarcity of prospective and randomized controlled trials that assess the impact of 3D modeling on concrete clinical outcomes such as complication rates, operative time, cost-effectiveness, or long-term functional results. Future research should aim to close these evidence gaps, especially in neonatal surgery, where clinical decisions are often made under conditions of anatomical complexity and time pressure. A standardized framework for study design, outcome reporting, and model validation would be instrumental in advancing the field toward evidence-based integration of 3D technology into surgical protocols. While early results are encouraging, randomized controlled trials assessing hard outcomes—morbidity, mortality, preserved organ function, total healthcare cost—are lacking. In conservative renal surgery for bilateral tumors, volumetric modeling seems advantageous. Yet there is no consensus on minimum viable renal volume or validated predictive algorithms for functional outcomes. Pratt et al. note that, in fetal surgery, despite growing use of computer-assisted planning, no direct neonatal outcome improvement has been demonstrated [22]. The same applies to EXIT, neonatal thoracoscopy, and congenital heart surgery, where 3D models are useful for surgeons but lack long-term outcome studies. The result of heterogeneity, lack of usage criteria, and absence of control group comparisons hinder generalizability and call for prospective, multicenter clinical research with standardized endpoints.

A further obstacle is unequal access. While some centers in North America, Europe, and Asia have in-house engineering units, most pediatric hospitals in Latin America, Africa, or Eastern Europe do not. This disparity threatens to widen technological inequities. In many countries, 3D modeling is neither regulated nor reimbursed, depending on team interest, foundations, or private support. While partnerships with external companies offer interim solutions, broader structural integration is needed. There is also a lack of formal recognition of the clinical and economic value of 3D models. Billing codes, regulatory standards, and clinical guidelines are still largely absent.

Despite these limitations, future prospects are promising. Advances in AI-based segmentation, multimodal image fusion, and extended reality platforms are setting the stage for broader, standardized adoption. AI tools based on deep learning will accelerate modeling and reduce operator variability. Pediatric anatomical libraries, developmental stage segmentation templates, and TC-MRI-ultrasound fusion protocols are already under development. Virtual and augmented reality platforms will expand access without requiring physical prints, lowering costs and enabling remote, multidisciplinary collaboration. Regulatory frameworks and international quality standards are expected to emerge, ensuring reproducibility and safety for pediatric clinical use. Scientific validation through multicenter trials will be essential to justify systematic inclusion in surgical protocols, training curricula, digital health platforms, and reimbursement systems. At present, our group is conducting two validation studies on the application of 3D modeling in pediatric oncologic surgery, with a particular focus on its use in the surgical planning of neuroblastoma resections. However, further studies are needed to provide evidence and validate the use of these models in neonatal surgery.

## 5. Conclusions

This article has comprehensively reviewed the current state of three-dimensional modeling applied to neonatal surgery, integrating international evidence, technical foundations, and clinical experience based on real cases. Three-dimensional modeling represents a transformative tool for addressing complex neonatal surgical conditions, with a direct impact on surgical planning, procedural safety, family communication, and clinical team training.

Although evidence remains limited, current data—reinforced by our clinical experience—indicate that both physical and virtual 3D models improve the understanding of complex anatomies, enable personalized surgical strategies, and support safer, more conservative, and better-planned interventions. Beyond the surgical act, 3D modeling is a valuable tool for medical education and family communication. In pediatric contexts, where anatomical variability and family involvement are critical, these models create new opportunities for dialogue, fostering shared decision-making and reducing emotional and cognitive uncertainty.

This technology should be considered an important complement in the planning of complex cases, such as congenital heart malformations, thoracic anomalies, or retroperitoneal tumors. Its effective use requires collaboration between institutions and specialized providers, as well as the establishment of dedicated 3D modeling units integrated with imaging, surgery, pathology, and education. Furthermore, robust multicenter research and clear technical standards—covering segmentation, validation, and traceability—are essential to ensure consistent quality and equitable clinical application.

Equitable and sustainable access to this technology must also be prioritized, which involves incorporating it into public health policies, institutional funding strategies, and professional training programs. Documenting real-world experiences, such as those described in this article, is essential to validate, refine, and project the true clinical impact of 3D modeling in pediatric practice. We consider the use of 3D modeling in neonatal surgical planning and standard surgical workflows to be an innovative approach with significant clinical potential.

## Figures and Tables

**Table 1:** Summary of Clinical Cases Using 3D Modeling in Neonatal Surgery.

Case	Condition	Imaging Modalities	Model Type	Clinical Impact
**1**	Right-sided CDH	Fetal MRI	Digital 3D Model	Enhanced prognosis, improved planning, family counseling
**2**	CDH + CPAM	Fetal MRI	Digital 3D Model and 1:1 Printed Model	Emergency preparedness, spatial simulation
**3**	Craniopagus conjoined twins	Fetal MRI	Digital 3D Model and 1:1 Printed Model	Surgical planning for separation, clinical simulation
**4**	Thoraco-omphalopagus conjoined twins	MRI	Digital 3D Model and 1:1 Printed Model	Surgical planning for separation, clinical simulation
**5**	Cloacal malformation	CT Cloacogram	Digital 3D Model	Accurate anatomical mapping, technique selection
**6**	Bilateral neuroblastoma	MRI	Digital 3D Model	Pre-surgical vessel assessment, recurrence detection

## Data Availability

The original contributions presented in the study are included in the article; further inquiries can be directed to the corresponding author.
